# Amplicon Deep Sequencing Reveals Multiple Genetic Events Lead to Treatment Failure with Atovaquone-Proguanil in Plasmodium falciparum

**DOI:** 10.1128/aac.01709-22

**Published:** 2023-05-08

**Authors:** Daniel Castañeda-Mogollón, Noah B. Toppings, Claire Kamaliddin, Raynell Lang, Susan Kuhn, Dylan R. Pillai

**Affiliations:** a Cumming School of Medicine, Department of Pathology & Laboratory Medicine, the University of Calgary, Calgary, Alberta, Canada; b Cumming School of Medicine, Department of Microbiology, Immunology, and Infectious Diseases, the University of Calgary, Calgary, Alberta, Canada; c Calvin, Phoebe & Joan Snyder Institute for Chronic Diseases, the University of Calgary, Calgary, Alberta, Canada; d Cumming School of Medicine, Department of Medicine, the University of Calgary, Calgary, Alberta, Canada; e Cumming School of Medicine, Department of Community Health Sciences, the University of Calgary, Calgary, Alberta, Canada; f Cumming School of Medicine, Department of Pediatrics, the University of Calgary, Calgary, Alberta, Canada; g Alberta Precision Laboratories, Diagnostic & Scientific Centre, Calgary, Alberta, Canada

**Keywords:** *Plasmodium falciparum*, Malarone, amplicon deep sequencing, *de novo* mutation, complexity of infection, DNA sequencing, antimalarial agents, genomics, malaria

## Abstract

Atovaquone-proguanil (AP) is used as treatment for uncomplicated malaria, and as a chemoprophylactic agent against Plasmodium falciparum. Imported malaria remains one of the top causes of fever in Canadian returning travelers. Twelve sequential whole-blood samples before and after AP treatment failure were obtained from a patient diagnosed with P. falciparum malaria upon their return from Uganda and Sudan. Ultradeep sequencing was performed on the *cytb, dhfr,* and *dhps* markers of treatment resistance before and during the episode of recrudescence. Haplotyping profiles were generated using three different approaches: *msp2-3D7* agarose and capillary electrophoresis, and *cpmp* using amplicon deep sequencing (ADS). A complexity of infection (COI) analysis was conducted. *De novo cytb* Y268C mutants strains were observed during an episode of recrudescence 17 days and 16 h after the initial malaria diagnosis and AP treatment initiation. No Y268C mutant reads were observed in any of the samples prior to the recrudescence. SNPs in the *dhfr* and *dhps* genes were observed upon initial presentation. The haplotyping profiles suggest multiple clones mutating under AP selection pressure (COI > 3). Significant differences in COI were observed by capillary electrophoresis and ADS compared to the agarose gel results. ADS using *cpmp* revealed the lowest haplotype variation across the longitudinal analysis. Our findings highlight the value of ultra-deep sequencing methods in the understanding of P. falciparum haplotype infection dynamics. Longitudinal samples should be analyzed in genotyping studies to increase the analytical sensitivity.

## INTRODUCTION

The combination of atovaquone-proguanil (AP; brand name Malarone) is used as a chemoprophylactic agent against malaria and for treatment of uncomplicated malaria in Canada (1). The mechanism of action of atovaquone consists of the disruption of the electron transport chain at the *bc_1_* complex, resulting in the loss of mitochondrial function (2). The mechanism of proguanil consists of inhibiting dihydrofolate reductase (*dhfr*), which disrupts pyrimidine biosynthesis, and thus nucleic acid replication (3). Point mutations in the cytochrome b (*cytb*) gene of the parasite have been shown to confer resistance to atovaquone (4). In addition, parasites harboring *dhfr* mutations provide resistance to cycloguanil (the active metabolite of proguanil) and pyrimethamine (5). Similar to *dhfr*, the dihydropteroate synthase (*dhps*) gene encodes a protein involved in the folate pathway of nucleic acid synthesis (6). Mutations in the *dhps* gene are known to confer resistance to sulfadoxine (6).

The efficacy of AP has been recorded to vary between 89% to 98% for P. falciparum (7). Reasons behind AP treatment failure include drug malabsorption during administration of the drug without fatty food in subtherapeutic serum levels (8, 9) C/N/S mutants in the 268 *cytb* codon, insufficient dosage (10), and obesity (11).

*In vivo* treatment failure and *in vitro* resistance to AP has been widely observed in isolates with Y268 *cytb* mutations. Previous studies have recorded an atovaquone IC_50_ for wild-type (WT) parasites below 10 nM, while isolates with C,N, or S at position 268 vary between 20.5 and 17,000 nM (12–14). Furthermore, studies using animal models have provided evidence of positive selection of *cytb* Y268S/N/C in response to suboptimal doses of AP in P. berghei and P. yoelii induced infections (15, 16).

The global emergence and spread of multidrug-resistant clones represent a threat to malaria elimination (17). Genomic surveillance plays a critical component in tracking and identifying clones correlated with treatment failure. For this, haplotyping techniques have been developed with the aim of accurately assessing clonality, detecting the presence of minor clones, and designing drug efficacy trials. Traditional haplotyping techniques include the use of length polymorphic markers of the merozoite surface protein 1 (*msp1*), merozoite surface protein 2 (*msp2*), and the glutamate-rich protein (*glurp*), as well as a set of microsatellite markers to characterize haplotyping profiles (18). Next-generation sequencing (NGS) provides a more powerful alternative for haplotyping whole genomes or highly polymorphic amplicons (18).

Amplicon deep sequencing (ADS) has been widely used in identifying drug-resistant clones, screening for positive selection mutants (19), and assessing the complexity of infection (COI) in *Plasmodium* isolates. Previous studies have highlighted the advantages of ADS over traditional techniques for haplotyping and SNP calling, including its discriminatory power to identify haplotypes with a frequency as low as 0.1% and with low parasitemia levels (<5 copies/μL) (20–23). Additionally, highly heterozygous markers (conserved *Plasmodium* membrane protein–*cpmp*; circumsporozoite protein–*csp;* apical membrane antigen 1–*ama1;* serine repeat antigen 2–*SERA2*), have been successfully evaluated for adequate haplotype discrimination in mock and clinical samples, and thus, provide a better understanding of malaria infection dynamics, linkage disequilibrium, and epistasis (20, 21, 24).

**FIG 1:**
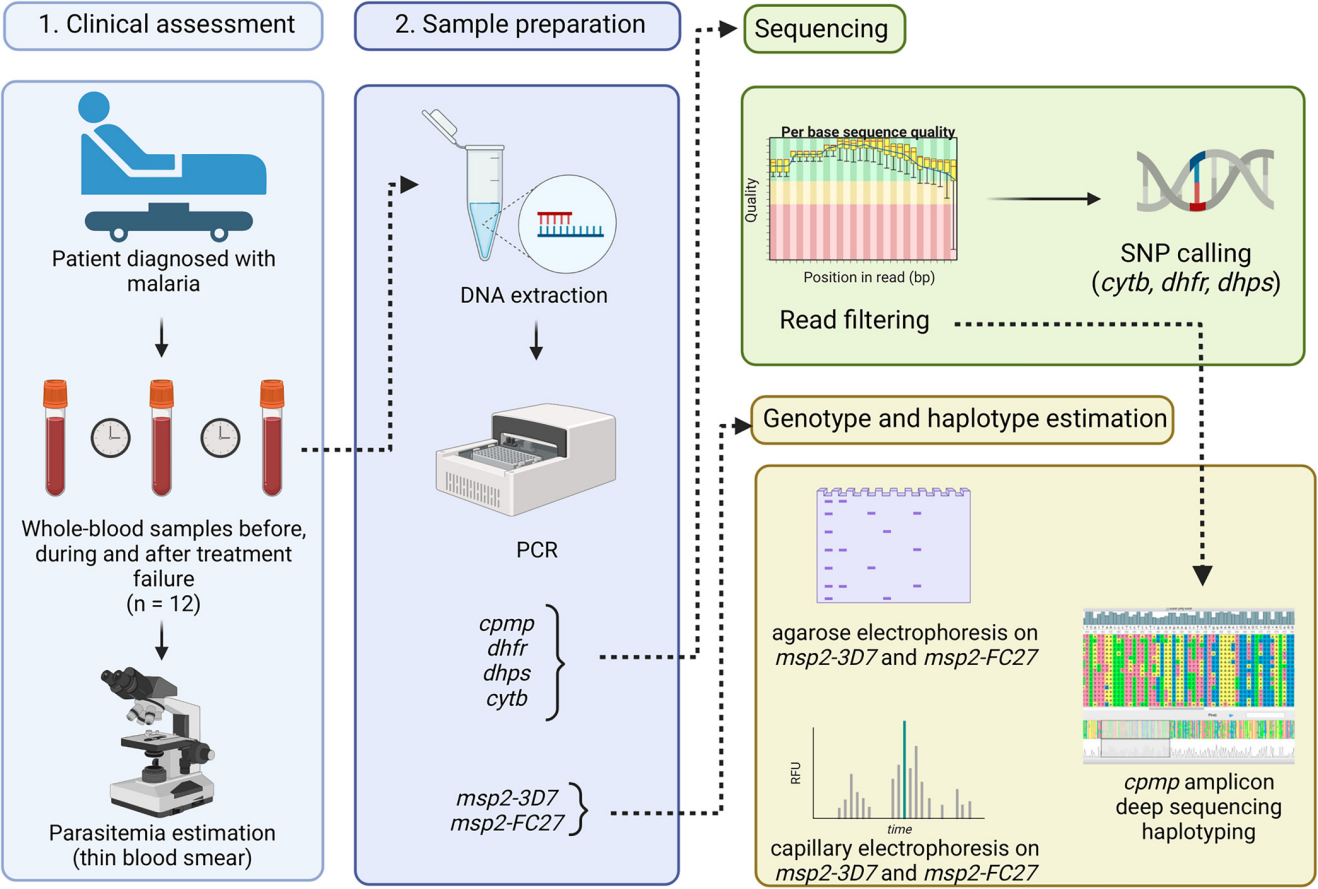
Haplotyping and SNP calling workflow. Whole blood samples (*n* = 12) were collected across the patient care. P. falciparum diagnostic and parasitemia was assessed using a Giemsa-stained thin blood smear. DNA was extracted from whole-blood samples followed by PCR amplification and Illumina sequencing of three genes associated with treatment failure in P. falciparum malaria (*cytb*, *dhfr*, and *dhps*). Haplotyping was performed using three different approaches by amplifying the following gene markers: *msp2-3D7*, *msp2-FC27*, and *cpmp*. SNP calling was performed using GATK. Traditional haplotyping approaches (2% agarose gel and capillary electrophoresis) were performed on *msp2-3D7* and *msp2-FC27*. Amplicon deep sequencing haplotyping was performed with *cpmp* using DADA2. This figure was created with BioRender.com.

In this study, we evaluated malaria infection dynamics and resistance haplotypes of a P. falciparum clinical isolate in a traveler returning from a 9-week trip to Uganda and Sudan. The patient presented an early treatment failure to AP for uncomplicated malaria. We first assessed the mutation profiles of *cytb*, *dhfr*, and *dhps* using an ultradeep sequencing approach. With these sequencing profiles, specific mutations known to confer drug resistance could be identified and evaluated before and after treatment failure. We then investigated the haplotyping profile across the infection course by using three techniques: (i) agarose gel haplotyping of the *msp2*, (ii) capillary electrophoresis of *msp2*, and (iii) ADS of *cpmp* (Fig. 1).

This study demonstrates AP early treatment failure caused by a *cytb* Y268C *de novo* mutation in Plasmodium falciparum. This was possible by employing an ultradeep sequencing approach to evaluate the P. falciparum *cytb* gene using a series of longitudinal samples.

## RESULTS

### Clinical background.

A 14-year-old Canadian female traveled to Uganda and South Sudan between June 16 and August 22 of 2019. She was prescribed AP (250 mg daily) by a health clinic prior to her trip. She was intermittently compliant with the chemoprophylaxis during the trip, missing a total of 3 weeks of AP over the approximately 9-week travel period. Three days after her return, the traveler developed a fever (38.2°C), headache, nausea, myalgia, and vomiting. On August 28 she was admitted to a hospital in Calgary, AB where she was diagnosed with P. falciparum infection by microscopy. The traveler, weighing 80.1 kg, was treated with a 3-day course of AP 18 h after the hospital admission. After the course of treatment, the patient was asymptomatic, and no parasites were observed. Eighteen days after her initial admission, and without subsequent travel, the patient was feeling unwell and readmitted to the hospital. She was febrile (40.2°C), developed headache, nausea, and vomiting. She was diagnosed with P. falciparum malaria after a blood smear revealed 2.6% parasitemia. Shortly after she was initiated on doxycycline (100 mg) and quinine sulfate (600 mg) for a 7-day course. She was discharged 2 days after her last admission with no further symptoms.

### Genomic markers of resistance to atovaquone-proguanil.

**FIG 2:**
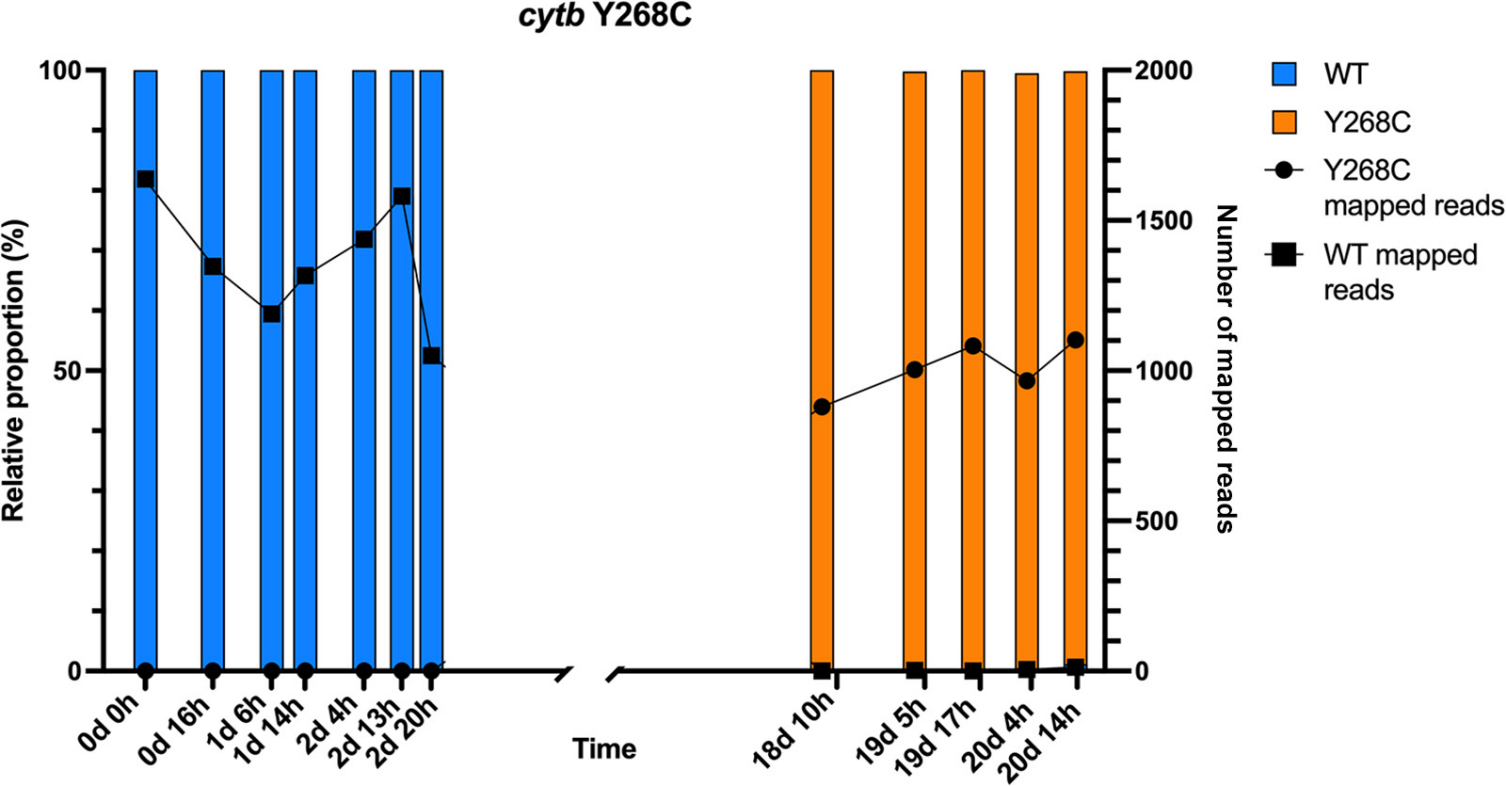
Relative proportion of the read depth of the wild type *cytb* Y268 (blue) and the *cytb* Y268C mutant (orange) over time.

A total of 622,605 reads were sequenced for the three resistance markers. A read depth average ± standard error of the mean (SE) per gene across all samples was computed, with a mean and SE depth of 1,719 ± 319.3 for *cytb*, 2,190 ± 865.7 for *dhfr*, and 1,864 ± 999.2 for *dhps*. SNP calling was performed, with SNPs called only if 100 reads mapped to a given position and at least 1% of reads contained a SNP. A total of 7 SNPs and 5 deletions were observed across the three genes of interest over the 12 clinical samples. Of these, four SNPs previously correlated with treatment resistance were observed, including the A803G (coding for Y268C) in *cytb* conferring resistance to atovaquone, c1790g (coding for A437G) in *dhps* conferring resistance to sulfadoxine, and the *dhfr* a343t (N51I) and g514t (S108N), known to result in resistance to cycloguanil (5) (Table 1). The *dhps* and *dhfr* SNPs associated with treatment resistance were observed across all 12 clinical samples (Fig. S1). The *cytb* mutant, on the other hand, was undetected across the seven samples prior to the treatment failure but became the dominant genotype during recrudescence. We thus found no evidence, within the limits of detection of this approach, that the variant was preselected and already present in the first malaria episode (Fig. 2). During the episode of recrudescence, three out of five samples had a few reads mapped to the *cytb* WT gene. A total of 3 WT reads (0.20%) were mapped to the 19d 5h sample, 6 WT reads were mapped to the 20d 4h sample, and 13 WT reads were mapped to the 20d 14h sample. Overall, the *cytb* sequencing profile indicates a fixation of the *cytb* Y268C at the time of treatment failure.

**TABLE 1:** Drug resistance mutations prevalence in *cytb*, *dhfr*, and *dhps*

Time point	*cytb* mutant and prevalence	*dhfr* mutant and prevalence	*dhps* mutants and prevalence
0d 0h (pretreatment)	WT (100%)	N51I (99.88%), S108N (99.88%)	A437G (100%)
0d 16h	WT (100%)	N51I (100%), S108N (99.21%)	A437G (100%)
1d 6h	WT (100%)	N51I (99.84%), S108N (100%)	A437G (100%)
1d 14h	WT (100%)	N51I (99.57%), S108N (100%)	A437G (100%)
2d 4h	WT (100%)	N51I (99.80%), S108N (100%)	A437G (100%)
2d 13h	WT (100%)	N51I (99.93%), S108N (100%)	A437G (100%)
2d 20h	WT (100%)	N51I (99.92%), S108N (99.92%)	A437G (99.92%)
18d 10h	Y268C (100%)	N51I (99.94%), S108N (100%)	A437G (100%)
19d 5h	Y268C (99.80%)	N51I (99.82%), S108N (100%)	A437G (99.92%)
19d 17h	Y268C (100%)	N51I (99.77%), S108N (100%)	A437G (100%)
20d 4h	Y268C (99.48%)	N51I (99.87%), S108N (100%)	A437G (100%)
20d 14h	Y268C (98.82%)	N51I (99.88%), S108N (100%)	A437G (99.93%)

Interestingly, the *dhps* c2879g UTR SNP was found at 100% prevalence in four of the seven samples before recrudescence (0 days 0 h, 1 day 6 h, 1 day 14 h, and 2 day 4 h), and two of the five samples during recrudescence (18 day 10 h, 19 day 5 h, 19 day 17 h) (Table S1). This pattern of alternating mutant prevalence was observed before and during recrudescence. No sequence variants in *cytb*, *dhfr*, or *dhps* were detected in the P. falciparum 3D7 positive control.

### Complexity of infection and haplotyping analysis.

**FIG 3:**
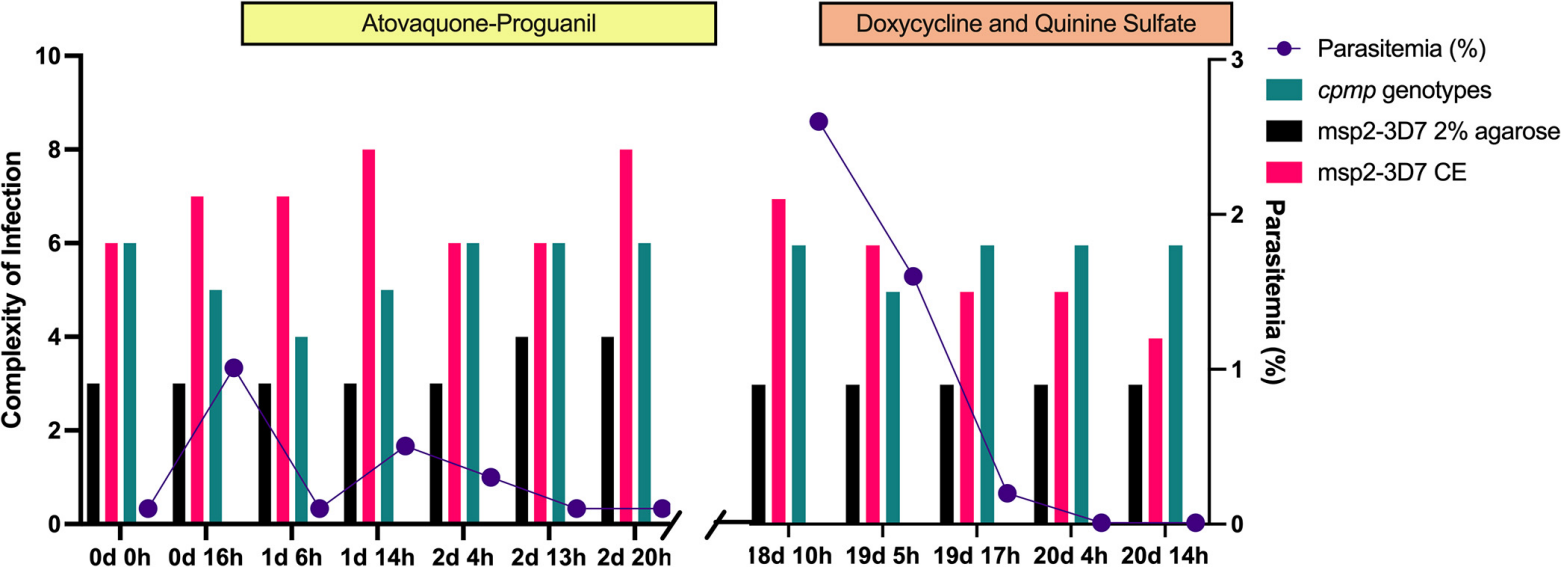
*De novo* emergence of *cytb* Y268C and haplotype profile. (a) Complexity of infection assessment across each blood sample by *msp2-3D7* (black), *msp2-3D7* capillary electrophoresis (pink), and *cpmp* haplotyping (teal) overlaid with the observed parasitemia (purple) over time. AP treatment (yellow) started 18 h after the first blood sample collection for the duration of 3 days. Quinine sulfate treatment (orange) started shortly after hospital readmission for the duration of 7 days.

To determine whether the *cytb* Y268C mutant observed during the treatment arose due to a *de novo* mutation or was simply a minor clone undetected during the first days of infection, we performed a haplotyping analysis using gel electrophoresis, capillary electrophoresis, and ADS (Fig. 3 and Table 2). This approach also allowed us to determine if the change in frequency of Y268C resulted in a soft or hard selection sweep.

**TABLE 2:** Haplotyping profiles of *msp2-3D7* and *cpmp*

	Haplotype profiles			
Time (days, hours)	Agarose electrophoresis *msp2-3D7* (bp)	Capillary electrophoresis *msp2-3D7* (bp)	ADS *cpmp* haplotype ID	Parasitemia (%)
0d 0h	412, 531, 668	353, 365, 371, 376, 383, 523	1,2,3,4,5,6	0.1
0d 16h	370, 531, 668	353, 365, 371, 376, 383, 389, 638	1,2,3,4,5	1
1d 6h	370, 505, 668	346, 353, 358, 365, 371, 376, 383	1,2,3,4	0.1
1d 14h	370, 505, 647	346, 353, 358, 365, 371, 376, 383, 516, 638	1,2,3,4,5	0.5
2d 4h	412, 552, 668	353, 365, 371, 376, 383, 523	1,2,3,4,5,6	0.3
2d 13h	370, 505, 531, 647	353, 365, 371, 376, 383, 523	1,2,3,4,5,6	0.1
2d 20h	370, 473, 531, 647	358, 365, 376, 383, 460, 516, 523, 638	1,2,3,4,5,6	0.1
18d 10h	412, 552, 668	353, 365, 371, 376, 383, 389, 523	1,2,3,4,5,6	2.6
19d 5h	370, 531, 668	353, 371, 376, 383, 389, 523	1,2,3,4,5	1.6
19d 17h	370, 473, 531	371, 383, 453, 460, 523	1,2,3,4,5,6	0.2
20d 4h	412, 552, 668	358, 376, 516, 523, 638	1,2,3,4,5,6	0.01
20d 14h	370, 552, 668	376, 383, 516, 523	1,2,3,4,5,6	0.01

The first method for haplotyping consisted of estimating the number and size of *msp2-3D7* and *msp2-FC27* haplotypes via agarose electrophoresis. All the samples amplified the *msp2* gene (*n* = 12). A range of 3 to 4 *msp2-3D7* haplotypes was observed prior to the recrudescence, and 3 haplotypes were observed after treatment failure. All haplotypes were observed before and during the episode of recrudescence. (Table S2 and Fig. S2). The *msp2-3D7* fragment sizes ranged from 370 bp to 668 bp. The 668 bp was the most prevalent haplotype across all samples. No *msp2-FC27* family-specific alleles were observed. None of the *msp2-3D7* haplotypes observed by this method were consistently found across all 12 samples.

Similar to the agarose electrophoresis, we estimated the number and size of *msp2-3D7* and *msp2-FC27* haplotypes using capillary electrophoresis. A range of 6 to 8 *msp2-3D7* haplotypes was observed across the samples prior to the treatment failure, and a range of 4 to 7 haplotypes was observed during the episode of recrudescence. The *msp2-3D7* fragment sizes ranged from 346 bp to 638 bp (Table S3). As seen with gel electrophoresis, no *msp2-FC27* family-specific alleles were observed. All haplotypes were observed before and during the recrudescence with the exception of one unique haplotype in an individual sample after the treatment failure (19 days 17 h). Similar to the agarose electrophoresis, none of the haplotypes were consistently observed across all samples. To determine the ability of this assay to detect minor haplotypes, we generated contrived samples of two haplotypes mixed at varying frequencies. We were able to detect the minor haplotype using >10 copies/μl of DNA for samples mixed 50%-50%, 75%-25%, and 90%-10%, and using >100 copies/μl of DNA for those mixed 95%-5% and 99%-1%.

**FIG 4:**
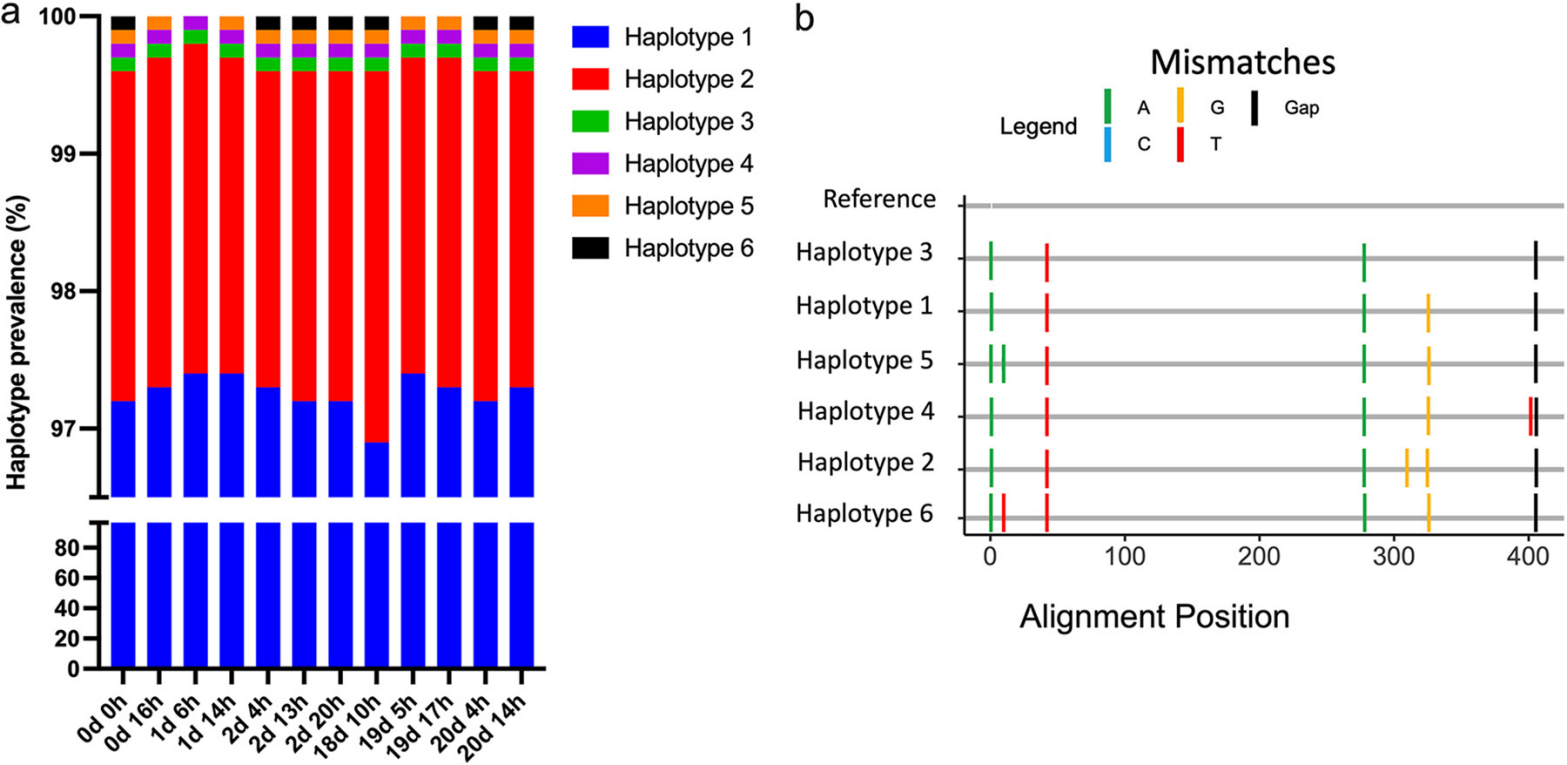
Haplotype profiles of the *cpmp* marker. (a) Relative proportion plot of each called haplotype before and during the episode of recrudescence. (b) Highlighter plot of the haplotype diversity compared to the P. falciparum *3D7 cpmp* gene (PlasmoDB: PF3D7_0104100).

The last approach consisted of the haplotype analysis of the *cpmp* gene using ADS (mean of 652,235 ± 29,417) (Fig. S4). Here, we observed a range of 4 to 6 *cpmp* haplotypes across the samples prior to the treatment failure, and a range of 5 to 6 haplotypes was observed during the recrudescence. All *cpmp* haplotypes were observed before and after recrudescence (Table S4). Four of the six haplotypes (66.66%) were found across all 12 samples. Overall, two major haplotypes were dominant across all samples, without any significant changes in their proportion, while the remaining four haplotypes were found at a lower prevalence (Fig. 4a). A range of 3 to 5 polymorphisms and a gap were observed between the haplotypes reported and the P. falciparum 3D7 *cpmp* reference gene (PlasmoDB: PF3D7_0104100) (Fig. 4b). Using single and mixed haplotype contrived controls, we observed a limit of detection of 0.1 copies/μl. No artifactual variants were observed in the control samples using the specified thresholds.

The three haplotyping profiles generated did not identify a minor clone that could have been preselected prior to the treatment failure. All haplotypes generated were identified before and during the episode of recrudescence (apart from one haplotype on 19d 17h by capillary electrophoresis), suggesting maintenance of genetic diversity during the episode of recrudescence, leading to a selective soft sweep.

None of the haplotypes observed in the clinical samples by agarose gel or capillary electrophoresis, nor the haplotypes estimated by the ADS approach were detected in the positive controls. This indicates *msp2* and *cpmp* are good markers for haplotype discrimination.

To investigate the performance of haplotyping approaches, we compared the COI by methods before and during the episode of recrudescence (Fig. S3). When comparing the COI values before the episode of recrudescence, we observed significantly different results between the agarose electrophoresis *versus* the capillary electrophoresis (Dunn’s adjusted *P* value = 0.01), the agarose electrophoresis *versus* the *cpmp* ADS (Dunn’s adjusted *P* value = 0.002), and no significant differences between the capillary electrophoresis *versus* the *cpmp* ADS (Dunn’s adjusted *P* value > 0.99). The COI values were also compared during the episode of recrudescence, with significantly different values between the agarose electrophoresis *versus* the capillary electrophoresis (Dunn’s adjusted *P* value = 0.04), agarose electrophoresis *versus* the *cpmp* ADS (Dunn’s adjusted *P* value = 0.01), and no significant differences between the capillary electrophoresis *versus* the *cpmp* ADS (Dunn’s adjusted *P* value > 0.99). Thus, the same COI trends across the methods were observed before and during recrudescence.

## DISCUSSION

To our knowledge, this is the first described observation of an early clinical failure of AP treatment caused by multiple *cytb* Y268C *de novo* mutations with distinct genetic backgrounds (COI > 3). In this study, three haplotyping approaches and a multilocus ADS approach were used to determine the genetic longitudinal profiles of 12 samples before and during an episode of recrudescence. Data presented in this work support prior reports of *de novo* Y268 *cytb* mutations in returning travelers and in malaria-endemic populations while being treated with AP (25–29).

The three methodologies agree on a similar haplotyping pattern across all samples, suggesting no clearance of any of the clones during the first 3 days of treatment. Additionally, no Y268C reads were observed during the seven samples prior to recrudescence, eliminating the possibility of an existing preselected mutant. The maintenance of genetic diversity by the haplotyping results suggests a soft selective sweep occurred within a window of 17 days and 16 h of at least four independent clones. Current studies highlighting AP treatment failure have previously identified one to two haplotypes independently acquiring the same *de novo* mutation (4, 14, 25–27, 30). No studies have shown evidence of multiclonal (COI > 3) *de novo* mutations at the 268th position of the *cytb* gene.

Despite having a consistent haplotype profile before and during the episode of recrudescence, the capillary and gel electrophoresis failed to identify a consistent haplotype across all 12 samples. ADS, on the other hand, was able to capture 4 out of 6 haplotypes consistently. Despite recording nonsignificant differences in COI by capillary electrophoresis and *cpmp* ADS, we observed a higher variability in haplotype calls from capillary electrophoresis. In addition, both the capillary electrophoresis and the ADS methodologies estimated a higher COI, suggesting a higher sensitivity as previously discussed (3, 7). Similar to our findings, patterns of unique haplotypes have been previously recorded in longitudinal studies using *msp1* and *msp2* as haplotyping markers (28). This suggests the COI in the parasite population could be underestimated when using individual samples. This is of paramount importance for therapeutic efficacy trials and genomic surveillance.

Current reports highlighting AP treatment failure are limited by using Sanger sequencing, agarose gel haplotyping of length-polymorphic markers, or a small sample size (4, 9, 14, 25, 29–32). This could underestimate the haplotype profile and/or the type of selective sweep in *de novo* mutations. Indeed, previous studies have highlighted the low mutant detectability of Sanger sequencing in contrived samples, suggesting a limit of detection as low as 70% (17). Additionally, length-polymorphic markers estimate haplotypes by looking at the size of alleles between treatment and follow-up samples. These methods have several limitations, such as the preferential amplification of shorter alleles, higher limits of detection (LOD), decreased specificity, and formation of spurious bands (21, 22, 33, 34). Despite this, recent studies still use length polymorphic markers (*msp1*, *msp2*, *glurp*) and microsatellites for haplotyping (35, 36). This could lead to a bias in their findings, especially if no posterior Bayesian correction for recrudescence or reinfection was applied or if a unique sample was taken at a given time (28, 37, 38).

Classical models of treatment failure consist of a beneficial allele being part of a population and expanding under directional drug selection (30). Remarkably, no *cytb* Y268C allele was detected in any of the samples prior to the recrudescence within the limit of detection of ADS. Because no parasitic recombination occurs during the erythrocytic stage, it is likely that the *cytb* Y268C arose independently in multiple clones with distinct genetic backgrounds while maintaining their genetic diversity after the treatment failure. An alternative explanation is that Y268C selection occurred due to a long period of subtherapeutic AP exposure that could have been competing with other genotypes under low AP pressure. It is possible but less likely that low levels of the mutant were not detected in ADS due to a skew in the read distribution by the 30 cycles of amplification during the library preparation. Furthermore, all the clinical samples from this study carried two *dhfr* mutations (S108N, N51I) that render resistance to cycloguanil (5), which have been reported in southwest Uganda (39). Amplicon deep sequencing protocols should be used to determine the nature of the selective sweep when evaluating the genetic emergence of resistance in *Plasmodium*, in order to understand the implications behind the propensity of AP resistance development.

Surprisingly, the time to recrudescence was shorter than the estimated intervals for AP therapeutic failure previously reported by Sutherland et al. (95% C.I 23 to 33 days) and by the metanalysis from Staines et al. (95% C.I. 22 to 35 days) (7, 40). Previous studies have observed early AP treatment failures but were not associated with a point mutation in the *cytb* gene (25, 41). Here, we hypothesize that subtherapeutic levels of atovaquone in the patient’s plasma from the chemoprophylactic agent prior to the first hospital admission led to increased selective pressure. This could have decreased the time period to for the symptoms to reappear. Indeed, suboptimal drug levels have been correlated with a atovaquone resistance in animal models (15).

Canadian guidelines for the treatment of uncomplicated P. falciparum consist of the administration of AP, or quinine and a second drug (e.g., doxycycline) (1). AP is a less favorable option to fixed-dose artemisinin combination treatments (ACTs) due to the risk of *de novo* resistance as observed in this study. Given the results reported in this study and other instances of treatment failure with AP in the literature, there is mounting evidence that revision of the treatment guidelines in Canada may be in order. However, access to alternative options need to be in place. Overall, this is the first study to observe an AP early treatment failure consisting of multiple P. falciparum haplotypes present before and during the episode of recrudescence. The data suggests a soft selective sweep of the *cytb* Y268C mutant that occurred 17 days after the initiation of treatment. No resistant mutants were observed in any of the samples prior to the episode of recrudescence. Amplicon deep sequencing is a useful tool in fully exploring the genetic emergence of resistance in P. falciparum.

## MATERIALS AND METHODS

### Included samples.

Twelve whole-blood samples were drawn and preserved at −80°C during patient care from a patient diagnosed with P. falciparum malaria returning from a 9-week trip to Uganda and South Sudan. The samples were collected before, during, and after treatment failure at day 0, 1, 2, 18, 19, and 20, and were subsequently analyzed for SNP calling and haplotyping (Fig. 1). Ethical approval was obtained from the Conjoint Health Research Ethics Board of the University of Calgary (REB15-1160). Patient written informed consent was obtained.

### DNA extraction, PCR, and sample purification.

DNA extraction was performed on frozen whole blood using the QIAamp DNA Blood minikit (Qiagen, Germany) following the manufacturer’s protocol. PCR was performed on a total of five genes: merozoite surface protein 2 (*msp2*), *cpmp*, *dhfr*, *dhps,* and *cytb*. The full-length *cytb*, *dhfr*, and *dhps* genes were amplified by adapting previous protocols (20, 42) (Tables S5 to 10). The *cpmp* PCR protocols were adapted from previous publications (20) (Tables S11 to 14).

The *msp2* gene was used for haplotyping using agarose gel electrophoresis and capillary electrophoresis, whereas the *cpmp* gene was used for ADS haplotyping. A primary PCR was performed on the *msp2* gene followed by a nested PCR on the 3D7 and FC27 alleles by adapting previous protocols (43) (Tables S15 to 20). The thermocycling conditions of the nested reaction were taken from the original protocol (43). The primer sequences for the primary and nested reactions were taken from the original protocol as well (43). The nested reaction was performed twice on each sample for the haplotyping analysis to prepare amplicons for agarose and capillary electrophoresis. For the latter, the 6-FAM fluorophore was added to the 5′ end of the reverse *3D7* and *FC27* primers.

The *cpmp*, *cytb*, *dhfr*, and *dhps* PCR products (referred to further as “amplicons”) were run on a 1% agarose gel for 1 h at 110 V. Each amplicon was visualized in the gel using GelRed Nucleic Acid Gel Stain and exposed to blue LED light in a Blue Light Transilluminator (Maestrogen). Amplicons were then and purified using the QIAquick Gel Extraction kit (Qiagen, Germany). Amplicons with a length below 1,000 bp were compared against a 100 bp ladder (Quick-Load Purple 100 bp DNA Ladder, NEB number N0551), while the remaining were compared against a 1 kb ladder (Quick-Load Purple 1 kb Plus DNA Ladder, NEB number N0550).

### Library preparation and sequencing.

The *cytb*, *dhfr*, and *dhps* amplicons of each sample were pooled in an equimolar fashion prior to the library preparation step. Two library preparations were generated. The first library preparation was performed across the *cytb*, *dhf*, and *dhps* amplicons by tagmenting using the Illumina DNA prep kit (Illumina, San Diego, CA, catalogue number 20060060) and barcoded with the Nextera XT v2 indexes (Illumina, catalogue number: FC-131-1024). The second library preparation was performed following the amplification of the *cpmp* gene by following Illumina 16S profiling protocol without tagmentation and barcoded with the Nextera XT v2 indexes (Illumina, catalogue number: FC-131-1024). Sequencing was carried out in two runs for each library using an Illumina MiSeq v3, 600 cycles, a 2% PhiX spike-in, and a maximum output of 25 million reads. A set of contrived samples was included in order to tune the software parameters for the *cpmp* ADS haplotype calling. This set consisted of six mixtures of the MRA-914 and MRA-1001 strains (100%:0%, 99%:1%, 95%:5%, 90%:10%, 75%:25%, 50%:50%) across six 10-fold dilutions (0.001 copies/μL to 100 copies/μL).

### Resistance markers SNP calling.

The sequencer fastq files were mapped to the corresponding resistance gene marker references from the PlasmoDB database (44) (*cytb*: PF3D7_0417200; *dhfr:* PF3D7_0417200; *dhps*: PF3D7_0810800) using the Burrows-Wheeler aligner (BWA) (45), followed by duplicate read flagging with Picard (46). The SNP calling parameters and file consolidation were adapted from previously validated methods (47). Briefly, SNP calling was performed with the Genome Analysis Toolkit (GATK) software (48) with SNPs called only if 100 reads mapped to a given position and at least 1% of reads contained a SNP with a quality Phred score of 30. File validation, manipulation, and indexing were done with Picard (46) and Samtools (49).

### Gel and capillary electrophoresis haplotyping.

Three techniques were employed to determine the haplotype profile before and after treatment failure: (i) agarose gel haplotyping on the family-specific alleles of *msp2*, (ii) capillary electrophoresis haplotyping of the family-specific alleles of *msp2*, and (iii) amplicon deep sequencing of the highly heterozygous *cpmp* gene marker.

The length-polymorphic *msp2-3D7* and *msp2-FC27* alleles were haplotyped using a 2% agarose gel and capillary electrophoresis. Both methods were adapted from previously described protocols (43). Briefly, the allele-specific amplicons were visually distinguished from each other based on fragment size assessed using a Quick-Load Purple 100 bp DNA Ladder (NEB number N0551) after being separated by a 2% agarose gel electrophoresis for 2 h at 110 V. The gels were visualized using a ChemiiDoc Touch Imaging System (Bio-Rad, version 2.3.0.07). The size and number of bands was estimated using Image Lab software (Bio-Rad, version 6.0.0). Alleles in each family were considered to be identical if the fragment sizes were within 20 bp of one another, as previously described (50, 51).

Capillary electrophoresis was performed using the GeneScan 1200 LIZ standard (Applied BioSystems) on a 3730 DNA Analyzer (Applied BioSystems) according to the manufacturer’s manual at the University of Guelph. Two replicates of each sample were run along with a negative control. In addition, a set of six mixtures of the MRA-914 and MRA-1001 strains (100%:0%, 99%:1%, 95%:5%, 90%:10%, 75%:25%, 50%,50%) across six 10-fold dilutions (0.001 copies/μL – 100 copies/μL) were used to determine the parameters for peak discrimination. To reduce false-positive calls from stutter peaks, companion peaks, and primers, four thresholds were applied: (i) allele-specific fragments below 200 bp were excluded (52); (ii) allele peaks within 3.3 bp of one another were considered to be the same (52); (iii) the allele peaks had to be present in both replicates within 0.4 bp of one another to be considered real; (iv) peaks below 550 relative fluorescent units (RFU) or below 20% of the highest peak were excluded. The analysis of the electropherograms was performed with the Open Source Independent Review and Interpretation System (OSIRIS) software (NCBI, version 2.16) (53).

### Amplicon deep sequencing haplotyping.

The *cpmp* gene marker was used for amplicon deep sequencing haplotyping using DADA2 (version 1.22) (54) implemented in R (version 4.1.3). The raw fastq files were demultiplexed. A Trimmomatic (55) sliding window of four nucleotides with a Phred score of <15 was used to remove low-quality reads. The primers of each read were trimmed with DADA2. The samples were filtered and truncated, followed by error rate estimation using the in-house DADA2 machine learning algorithm (54). Haplotype sample inference was performed on the filtered and trimmed data, followed by read merging and chimera removal with the RemoveBimeraDenovo function in DADA2. These putative haplotypes were then further filtered to prevent the call of false positives by excluding haplotypes (i) with a read depth below 1% (unless present in other independent samples at a minimum read depth of 0.1%), (ii) deviated from the expected nucleotide length of 430 bp, and (iii) a minimum of 100 reads. MAFFT (56) was used in l-INS-i mode to align the identified haplotypes. The complexity of infection was calculated and defined as the number of unique haplotypes within each independent sample.

### Statistical analysis and figures.

All the statistical analysis and figures were generated in GraphPad Prism (v9.4.1), Rstudio (v.1.0.153), and BioRender.

### Data availability.

Sequencing data have been deposited at the NCBI BioProject under the accession PRJNA875487. The electropherograms and raw gel picture are available at https://github.com/dcastaneda5/pillai_lab.git.
