# Supplementary material for: Amplicon Deep Sequencing Reveals Multiple Genetic Events Lead to Treatment Failure with Atovaquone-Proguanil in Plasmodium falciparum
Source: Antimicrob Agents Chemother. 2023 May 8;67(6):e01709-22. doi: 10.1128/aac.01709-22 (PMC10269153; doi:10.1128/aac.01709-22)
Supplement: Supplemental file 1 — Supplemental material. Download aac.01709-22-s0001.docx, DOCX file, 3.0 MB [file aac.01709-22-s0001.docx]

**Amplicon Deep Sequencing Reveals Multiple *Plasmodium falciparum* Genetic Events Leading to an Early Treatment Failure of Atovaquone-Proguanil**

Daniel Castañeda-Mogollón, Noah B. Toppings, Raynell Lang, Susan Kuhn, Claire Kamaliddin, Dylan R. Pillai

Supplementary figures

**Supplementary figure 1.** Number of raw reads sequenced from the *cytb* (average of 7,258 reads)*, dhfr* (average of 21,533 reads)*,* and *dhps* (average of 23,092 reads) genes known to confer treatment resistance.

**Supplementary figure 2.** Relative proportion of the read depth of the mutant (a) *dhfr* N51I, (b) *dhfr* S108N, and (c) *dhps* G437A.

**Supplementary figure 3.** *Msp2-3D7* PCR products run on 2% agarose gel for 2 hours at 110 V. L: 100 bp ladder (NEB #N0551), S1 to S13 indicate the samples analyzed before (S1) and during treatment (S2-S13). S1: 0d 0h (sample from the initial diagnosis); S2: 0d 9h; S3; 0d 16h, S4; 1d 6h; S5: 1d 14h; S6: 2d 4h; S7: 2d 13h; S8: 2d 20h; S9: 18d 10h; S10: 19d 5h; S11: 19d 17h; S12: 20d 4h; S13: 20d 14h; +: *P. falciparum 3D7* polyclonal control; -: Negative control extracted from a healthy donor; NFW: Negative nuclease-free water control.

**Supplementary figure 4**. Number of raw reads sequenced from *cpmp* heterozygous marker before and during the episode of recrudescence with an average mean of 652,235 reads.

**Supplementary figure 5.** Complexity of infection assessment by *msp2-3D7* agarose gel electrophoresis, *msp2-3D7* capillary electrophoresis, and *cpmp* haplotyping before (purple) and during (blue) the episode of recrudescence (* = *p-value* < 0.05; ** = *p-value* < 0.01; ns = not significant).

**Supplementary tables**

**Supplementary table 1. Master mix composition for the *cytb* PCR amplification.**

| PCR component | Volume |
| --- | --- |
| Collibri™ Library Amplification Master Mix (Thermo Fisher Scientific, catalogue number A38539250) | 11 µL |
| Forward primer (5’-CTATTAATTTAGTTAAAGCACAC-3’) | 1.25 µL [500 nM] |
| Reverse primer (5’-ACAGAATAATCTCTAGCACCA-3’) | 1.25 µL [500 nM] |
| Nuclease-free water | 6.50 µL |
| DNA template | 5 µL |

**Supplementary table 2. Thermocycling conditions for the *cytb* PCR amplification.**

| **Step** | **Temperature** | **Time** | **Number of cycles** |
| --- | --- | --- | --- |
| Initial denaturation | 98ºC | 3:00 min | 1 |
| Denaturation | 98ºC | 0:10 min | 30 |
| Annealing | 60ºC | 0:30 min |  |
| Extension | 65ºC | 3:00 min |  |
| Final extension | 65ºC | 10:00 min | 1 |

**Supplementary table 3. Master mix composition for the *dhfr* PCR amplification.**

| PCR component | Volume |
| --- | --- |
| Collibri™ Library Amplification Master Mix (Thermo Fisher Scientific, catalogue number A38539250) | 11 µL |
| Forward primer (5’- TTTTTACTAGCCATTTTTGTATTCC-3’) | 1.25 µL [500 nM] |
| Reverse primer (5’-TTAACCGTTCAGGTAATTTTGTCA-3’) | 1.25 µL [500 nM] |
| Nuclease-free water | 6.50 µL |
| DNA template | 5 µL |

**Supplementary table 4. Thermocycling conditions for the *dhfr* PCR amplification.**

| **Step** | **Temperature** | **Time** | **Number of cycles** |
| --- | --- | --- | --- |
| Initial denaturation | 98ºC | 3:00 min | 1 |
| Denaturation | 98ºC | 0:30 min | 30 |
| Annealing | 60ºC | 0:30 min |  |
| Extension | 65ºC | 5:00 min |  |
| Final extension | 65ºC | 10:00 min | 1 |

**Supplementary table 5. Master mix composition for the *dhps* PCR amplification.**

| PCR component | Volume |
| --- | --- |
| Collibri™ Library Amplification Master Mix (Thermo Fisher Scientific, catalogue number A38539250) | 11 µL |
| Forward primer (5’- AATATTTGCGCCAAACTTTTTA-3’) | 1.25 µL [500 nM] |
| Reverse primer (5’- TTTATTTCGTAATAGTCCACTTTTGAT-3’) | 1.25 µL [500 nM] |
| Nuclease-free water | 6.50 µL |
| DNA template | 5 µL |

**Supplementary table 6. Thermocycling conditions for the *dhps* PCR amplification.**

| **Step** | **Temperature** | **Time** | **Number of cycles** |
| --- | --- | --- | --- |
| Initial denaturation | 98ºC | 3:00 min | 1 |
| Denaturation | 98ºC | 0:30 min | 30 |
| Annealing | 58ºC | 0:30 min |  |
| Extension | 65ºC | 5:00 min |  |
| Final extension | 65ºC | 10:00 min | 1 |

**Supplementary table 7. Master mix composition for the *cpmp* primary amplification.**

| PCR component | Volume |
| --- | --- |
| Collibri™ Library Amplification Master Mix (Thermo Fisher Scientific, catalogue number A38539250) | 11 µL |
| Forward primer (5’-CGATACAGGACATATAGA -3’) | 1.25 µL [500 nM] |
| Reverse primer (5’-TTCAATAACATTTACTAGG -3’) | 1.25 µL [500 nM] |
| Nuclease-free water | 6.50 µL |
| DNA template | 5 µL |

**Supplementary table 8. Thermocycling conditions for the *cpmp* primary amplification.**

| **Step** | **Temperature** | **Time** | **Number of cycles** |
| --- | --- | --- | --- |
| Initial denaturation | 95ºC | 3:00 min | 1 |
| Denaturation | 98ºC | 0:20 min | 35 |
| Annealing | 49ºC | 0:15 min |  |
| Extension | 72ºC | 0:45 min |  |
| Final extension | 72ºC | 2:00 min | 1 |

**Supplementary table 9. Master mix composition for the *cpmp* nested amplification.**

| PCR component | Volume |
| --- | --- |
| Collibri™ Library Amplification Master Mix (Thermo Fisher Scientific, catalogue number A38539250) | 11 µL |
| Forward primer (5’-CATAAGTCATTAAAATTTATGGAT -3’) | 1.25 µL [500 nM] |
| Reverse primer (5’- CGTTACTATCAAGATCGTTAATATC-3’) | 1.25 µL [500 nM] |
| Nuclease-free water | 10.25 µL |
| PCR product from the primary reaction | 1.25 µL |

**Supplementary table 10. Thermocycling conditions for the *cpmp* nested amplification.**

| **Step** | **Temperature** | **Time** | **Number of cycles** |
| --- | --- | --- | --- |
| Initial denaturation | 95ºC | 3:00 min | 1 |
| Denaturation | 98ºC | 0:20 min | 20 |
| Annealing | 49.7ºC | 0:15 min |  |
| Extension | 72ºC | 0:45 min |  |
| Final extension | 72ºC | 2:00 min | 1 |

**Supplementary table 11. Master mix composition for the *msp2* primary amplification.**

| PCR component | Volume |
| --- | --- |
| Collibri™ Library Amplification Master Mix (Thermo Fisher Scientific, catalogue number A38539250) | 22 µL |
| Forward primer (5’-ATGAAGGTAATTAAAACATTGTCTATTATA-3’) | 2.50 µL [500 nM] |
| Reverse primer (5’- CTTTGTTACCATCGGTACATTCTT-3’) | 2.50 µL [500 nM] |
| Nuclease-free water | 13 µL |
| DNA template | 10 µL |

**Supplementary table 12. Thermocycling conditions for the *msp2* primary amplification.**

| **Step** | **Temperature** | **Time** | **Number of cycles** |
| --- | --- | --- | --- |
| Initial denaturation | 95ºC | 5:00 min | 1 |
| Denaturation | 94ºC | 1:00 min | 35 |
| Annealing | 59ºC | 2:00 min |  |
| Extension | 72ºC | 2:00 min |  |
| Final extension | 72ºC | 5:00 min | 1 |

**Supplementary table 13. Master mix composition for the *msp2-3D7* nested amplification.**

| PCR component | Volume |
| --- | --- |
| Collibri™ Library Amplification Master Mix (Thermo Fisher Scientific, catalogue number A38539250) | 11 µL |
| Forward primer (5’- AGAAGTATGGCAGAAAGTAAKCCTYCTACT-3’) | 1.25 µL [500 nM] |
| Reverse primer (5’-GATTGTAATTCGGGGGATTCAGTTTGTTCG-3’)^a^ | 1.25 µL [500 nM] |
| Nuclease-free water | 10.25 µL |
| PCR product from the primary reaction | 1.25 µL |

^a^ 6-FAM was attached in the in the 5’-end of the reverse primers for the capillary electrophoresis assay.

**Supplementary table 14. Thermocycling conditions for the *msp2-3D7* nested amplification**

| **Step** | **Temperature** | **Time** | **Number of cycles** |
| --- | --- | --- | --- |
| Initial denaturation | 95ºC | 5:00 min | 1 |
| Denaturation | 94ºC | 0:30 min | 30 |
| Annealing | 58ºC | 1:00 min |  |
| Extension | 72ºC | 1:00 min |  |
| Final extension | 72ºC | 5:00 min | 1 |

**Supplementary table 15. Master mix composition for the *msp2-FC27* nested amplification.**

| PCR component | Volume |
| --- | --- |
| Collibri™ Library Amplification Master Mix (Thermo Fisher Scientific, catalogue number A38539250) | 11 µL |
| Forward primer (5’-AATACTAAGAGTGTAGGTGCARATGCTCCA-3’) | 1.25 µL [500 nM] |
| Reverse primer (5’-TTTTATTTGGTGCATTGCCAGAACTTGAAC-3’)^a^ | 1.25 µL [500 nM] |
| Nuclease-free water | 10.25 µL |
| PCR product from the primary reaction | 1.25 µL |

^a^ 6-FAM was attached in the in the 5’-end of the reverse primers for the capillary electrophoresis assay.

**Supplementary table 16. Thermocycling conditions for the *msp2-FC27* nested amplification.**

| **Step** | **Temperature** | **Time** | **Number of cycles** |
| --- | --- | --- | --- |
| Initial denaturation | 95ºC | 5:00 min | 1 |
| Denaturation | 94ºC | 0:30 min | 30 |
| Annealing | 58ºC | 1:00 min |  |
| Extension | 72ºC | 1:00 min |  |
| Final extension | 72ºC | 5:00 min | 1 |

**Supplementary table 17. Mutant relative proportion *dhps* resistance marker before and during the episode of recrudescence.** Each column depicts the mutant proportion of each nucleotide position along with its prevalence compared to the *P. falciparum 3D7 dhps* (PF3D7) reference. Nucleotides in red depict a non-synonymous mutation known to cause sulfadoxine treatment failure. Nucleotides in green match the reference gene.

^a^: Nucleotide position found in an intronic region.

^b^: Nucleotide position found in an exonic region.

|  | *dhps* nucleotide position and prevalence (%) | | | | | | | |  |
| --- | --- | --- | --- | --- | --- | --- | --- | --- | --- |
| Time point | A^a^  **173** | A^a^  **488** | G^b^  **906** | C^b^  **1494** | G^b^  **1790** | T^a^  **2777** | T^a^  **2779** | C^a^  **2879** | **Mapped reads** |
| 0d 0h (pre-treatment) | del (91.85%) | del (97.42%) | A (100%) | A (100%) | C (100%) | del (86.49%) | del (98.36%) | G (100%) | 26,890 |
| 0d 16h | del (97.24%) | del (96.43%) | A (99.91%) | A (100%) | C (100%) | del (86.90%) | del (98.80%) | C (100%) | 22,392 |
| 1d 6h | del (90.43%) | del (97.48%) | A (99.92%) | A (100%) | C (100%) | del (86.49%) | del (97.24%) | G (100%) | 27,898 |
| 1d 14h | del (93.86%) | del (97.05%) | A (99.92%) | A (100%) | C (100%) | del (86.49%) | del (97.24%) | G (100%) | 21,343 |
| 2d 4h | del (93.60%) | del (97.64%) | A (100%) | A (99.91%) | C (100%) | del (82.79%) | del (97.93%) | C (100%) | 18,577 |
| 2d 13h | del (92.21%) | del (95.50%) | A (100%) | A (99.92%) | C (100%) | del (82.56%) | del (98%) | C (100%) | 23,288 |
| 2d 20h | del (94.93%) | del (93.44%) | A (100%) | A (99.92%) | C (99.92%) | del (87.77%) | del (99.21%) | C (100%) | 25,483 |
| 18d 10h | del (95.51%) | del (98.86%) | A (100%) | A (99.92%) | C (100%) | del (88.21%) | del (97.46%) | G (100%) | 24,733 |
| 19d 5h | del (91.11%) | del (91.95%) | A (99.80%) | A (99.92%) | C (99.92%) | del (80.33%) | del (97.46%) | G (100%) | 19,585 |
| 19d 17h | del (95.92%) | del (96.22%) | A (99.94%) | A (99.86%) | C (100%) | del (84.02%) | del (99.17%) | G (100%) | 23,174 |
| 20d 4h | del (93.90%) | del (82.35%) | A (99.93%) | A (99.91%) | C (100%) | del (83.14%) | del (97.53%) | C (100%) | 26,698 |
| 20d 14h | del (93.20%) | del (97.94%) | A (99.94%) | A (99.93%) | C (99.93%) | del (87.61%) | del (98.21%) | C (100%) | 17,045 |

**Supplementary table 18.** Haplotyping profile of the *msp2-3D7* by 2% agarose electrophoresis. An ‘X’ depicts the present of that haplotype at a particular point in time, a ‘-‘ represents the absecnce of that haplotype.

| **msp2-3D7**  **2% agarose gel** |  |  |  | |  |  | |  |  | |  | |  | |  | |  | |  | |  | |
| --- | --- | --- | --- | --- | --- | --- | --- | --- | --- | --- | --- | --- | --- | --- | --- | --- | --- | --- | --- | --- | --- | --- |
| **Haplotype (bp)** | **0d0h** | **0d 16h** | | **1d 6h** | **1d 14h** | | **2d 4h** | **2d 13h** | | **2d 20h** | | **18d 10h** | | **19d 5h** | | **19d 17h** | | **20d 4h** | | **20d 14h** | |  |
| **370** | - | X | | X | X | | - | X | | X | | - | | X | | X | | - | | X | |  |
| **412** | X | - | | - | - | | X | - | | - | | X | | - | | - | | X | | - | |  |
| **473** | - | - | | - | - | | - | - | | X | | - | | - | | X | | - | | - | |  |
| **505** | - | - | | X | X | | - | X | | - | | - | | - | | - | | - | | - | |  |
| **531** | X | X | | - | - | | - | X | | X | | - | | X | | X | | - | | - | |  |
| **552** | - | - | | - | - | | X | - | | - | | X | | - | | - | | X | | X | |  |
| **647** | - | - | | - | X | | - | X | | X | | - | | - | | - | | - | | - | |  |
| **668** | X | X | | X | - | | X | - | | - | | X | | X | | - | | X | | X | |  |
| **COI** | 3 | 3 | | 3 | 3 | | 3 | 4 | | 4 | | 3 | | 3 | | 3 | | 3 | | 3 | |  |

**Supplementary table 19.** Haplotyping profile of the *msp2-3D7* by capillary electrophoresis. An ‘X’ depicts the present of that haplotype at a particular point in time, a ‘-’ represents the absence of that haplotype. Haplotypes only found once across all samples were shown with a red ‘X’.

| **msp2-3D7-CE** |  |  |  | |  |  | |  |  | |  | |  | |  | |  | |  | |  | |
| --- | --- | --- | --- | --- | --- | --- | --- | --- | --- | --- | --- | --- | --- | --- | --- | --- | --- | --- | --- | --- | --- | --- |
| **Haplotype (bp)** | **0d0h** | **0d 16h** | | **1d 6h** | **1d 14h** | | **2d 4h** | **2d 13h** | | **2d 20h** | | **18d 10h** | | **19d 5h** | | **19d 17h** | | **20d 4h** | | **20d 14h** | |  |
| **346** | - | - | | X | X | | - | - | | - | | - | | - | | - | | - | | - | |  |
| **353** | X | X | | X | X | | X | X | | - | | X | | X | | - | | - | | - | |  |
| **358** | - | - | | X | X | | - | - | | X | | - | | - | | - | | X | | - | |  |
| **365** | X | X | | X | X | | X | X | | X | | X | | - | | - | | - | | - | |  |
| **371** | X | X | | X | X | | X | X | | - | | X | | X | | X | | - | | - | |  |
| **376** | X | X | | X | X | | X | X | | X | | X | | X | | - | | X | | X | |  |
| **383** | X | X | | X | X | | X | X | | X | | X | | X | | X | | - | | X | |  |
| **389** | - | X | | - | - | | - | - | | - | | X | | X | | - | | - | | - | |  |
| **453** | - | - | | - | - | | - | - | | - | | - | | - | | **X** | | - | | - | |  |
| **460** | - | - | | - | - | | - | - | | X | | - | | - | | X | | - | | - | |  |
| **516** | - | - | | - | X | | - | - | | X | | - | | - | | **-** | | X | | X | |  |
| **523** | X | - | | - | - | | X | X | | X | | X | | - | | X | | X | | X | |  |
| **638** | - | X | | - | X | | - | - | | X | | - | | X | | - | | X | | - | |  |
| **COI** | 6 | 7 | | 7 | 9 | | 6 | 6 | | 8 | | 7 | | 6 | | 5 | | 5 | | 4 | |  |

**Supplementary table 20.** Haplotyping profile of the *cpmp* by amplicon deep sequencing. An ‘X’ depicts the present of that haplotype in a particular point in time, a ‘-’ represents the absence of that haplotype.

| **cpmp ADS** |  |  |  |  |  | | |  | |  | |  | |  |  | |  | |  | |  | |
| --- | --- | --- | --- | --- | --- | --- | --- | --- | --- | --- | --- | --- | --- | --- | --- | --- | --- | --- | --- | --- | --- | --- |
| **Haplotype ID** | **0d0h** | **0d16h** | **1d 6h** | **1d14h** | | | **2d 4h** | | **2d13h** | | **2d20h** | | **18d10h** | | | **19d5h** | | **19d17h** | | **20d4h** | | **20d14h** |
| **1** | X | X | X | X | | X | | | X | | X | | X | | | X | | X | | X | | X |
| **2** | X | X | X | X | | X | | | X | | X | | X | | | X | | X | | X | | X |
| **3** | X | X | X | X | | X | | | X | | X | | X | | | X | | X | | X | | X |
| **4** | X | X | X | X | | X | | | X | | X | | X | | | X | | X | | X | | X |
| **5** | X | X | - | X | | X | | | X | | X | | X | | | X | | X | | X | | X |
| **6** | X | - | - | - | | X | | | X | | X | | X | | | - | | X | | X | | X |
| **COI** | 6 | 5 | 4 | 5 | | 6 | | | 6 | | 6 | | 6 | | | 5 | | 6 | | 6 | | 6 |
